# Integrated Morpho-Physiological and Biochemical Markers Rank Wheat Genotypes for Salinity and Drought Tolerance at the Seedling Stage

**DOI:** 10.3390/plants15060930

**Published:** 2026-03-18

**Authors:** Nimra Batool, Muhammad Yousaf Shani, Muhammad Yasin Ashraf, Samia Ahmad, Mazher Rasheed, Iman Fatima, Muhammad Azmat, Robina Aziz, Syed Mohsin Abbas, Ghulam Farid, William L. Bauerle

**Affiliations:** 1 Institute of Molecular Biology and Biotechnology (IMBB), The University of Lahore, Lahore 54000, Pakistan; nimraamalik83@gmail.com (N.B.); samiauol436@gmail.com (S.A.); muhammadazmat557@gmail.com (M.A.); 2 Nuclear Institute for Agriculture and Biology/College (NIAB-C), Pakistan Institute of Engineering and Applied Sciences (PIEAS), Islamabad 45650, Pakistan; mmyousafshani@gmail.com (M.Y.S.); imanadeel143@gmail.com (I.F.); faridniab@yahoo.com (G.F.); 3 Department of Molecular Biology and Genetics, Faculty of Engineering and Natural Sciences, Biruni University, Istanbul 34010, Türkiye; 4 Institute of Botany, University of the Punjab, Lahore 54000, Pakistan; mazharriazkhan735@gmail.com; 5 Department of Botany, Government College Women University Sialkot, Sialkot 51310, Pakistan; robinaaziz347@gmail.com; 6 Department of Horticulture, Faculty of Agricultural Sciences, University of Punjab, Lahore 54590, Pakistan; mohsinabbaspu@gmail.com; 7 Department of Horticulture and Landscape Architecture, Colorado State University, Fort Collins, CO 80523, USA

**Keywords:** antioxidant enzymes, osmolytes, MGIDI, PCA

## Abstract

Salinity and drought are major constraints to wheat productivity, affecting growth, photosynthesis, and cellular homeostasis. While many studies have examined responses to these stresses individually, comparative evaluation of genotypes under both stresses using an integrated physiological, biochemical, and multivariate framework remains limited. Here, six wheat genotypes were evaluated at the seedling stage under controlled salinity and drought treatments to identify key morphological and physio-biochemical markers associated with stress resilience. Both stresses reduced shoot and root growth, biomass, gas exchange, and photosynthetic pigments, with drought causing stronger inhibition. Among genotypes, Akbar-2019 exhibited the greatest tolerance, maintaining higher growth, pigment stability, photosynthetic performance, and membrane integrity, whereas Subhani-2021 was the most sensitive. Stress-induced osmotic adjustment was evident from increased proline, soluble sugars, and free amino acids, particularly in Akbar-2019. Antioxidant enzymes (SOD, POD, CAT, APX) were elevated under both stresses; Akbar-2019 combined stronger antioxidant activity with lower malondialdehyde and hydrogen peroxide levels, indicating effective mitigation of oxidative damage. Multivariate analyses (PCA, heatmap clustering, and MGIDI) consistently ranked Akbar-2019 as the most resilient genotype. These findings provide a novel, integrative framework for screening wheat under multiple abiotic stresses, identify promising genotypes for breeding and cultivation in stress-prone environments, and highlight combined morpho-physiological stability, osmolyte accumulation, and antioxidant capacity as informative markers for stress tolerance.

## 1. Introduction

Wheat (*Triticum aestivum* L.) is a major cereal crop worldwide and a staple in Pakistan, where it underpins food security and rural livelihoods [1]. Despite its importance, national yields remain below global averages, largely because drought and soil salinity frequently constrain crop establishment and productivity [2]. Globally, wheat is cultivated on ~218 million ha and contributes substantially to human caloric intake [3]. In Pakistan, wheat occupies ~9.0 million hectares and is grown by a large proportion of farmers as a primary income source. Meeting projected food demand for a population approaching 9.7 billion by 2050 will require sustained yield gains under increasingly variable climatic environments [4]. However, both biotic and abiotic stresses continue to limit wheat productivity [5]. Accordingly, screening wheat germplasm for drought- and salinity-tolerant phenotypes using robust morphological, physiological, and biochemical markers remains a practical route to accelerate genetic improvement [6].

Salinity stress affects a significant proportion of irrigated agricultural lands and severely restricts plant growth and productivity by disturbing physiological and metabolic processes. Elevated concentrations of sodium (Na^+^) and chloride (Cl^−^) ions disrupt cellular ion homeostasis and interfere with the uptake of essential nutrients such as potassium (K^+^) and calcium (Ca^2+^), ultimately impairing metabolic functioning and growth [7,8,9]. In addition, salinity reduces soil and leaf water potential, imposing osmotic stress and limiting water uptake, thereby generating physiological drought conditions even when soil moisture is present [10]. Comprehensive reviews of plant responses to salinity emphasize that successful tolerance mechanisms rely on efficient ion exclusion, compartmentalization of toxic ions, maintenance of osmotic balance, and preservation of photosynthetic activity [11,12].

Drought can reduce yield substantially by disrupting plant water status, reducing chlorophyll content, and impairing photosynthetic function [13,14]. Salinity inhibits germination, growth, and yield, and constrains gas exchange by reducing chlorophyll and carotenoids, altering chloroplast structure and photosynthetic electron transport, and decreasing stomatal conductance (gs) [15,16]. Both drought and salinity promote reactive oxygen species (ROS) formation, which, although functioning as signaling molecules at low concentrations, can cause lipid peroxidation, membrane injury, and damage to proteins and nucleic acids when accumulated excessively [17,18]. These stresses also perturb cellular metabolism, including compounds involved in osmotic protection (e.g., free amino acids, proline, soluble proteins, and sugars), thereby impairing physiological performance [19].

Identifying tolerant genotypes therefore requires integrative assessment of traits linked to stress resilience across scales. Osmotic adjustment via compatible solutes (proline, soluble sugars, and free amino acids) helps maintain hydration and cellular integrity under water and salt stress [20,21,22]. In parallel, enzymatic antioxidants including superoxide dismutase (SOD), peroxidase (POD), catalase (CAT), and ascorbate peroxidase (APX) are central to ROS detoxification and can serve as biochemical markers differentiating tolerant and sensitive germplasm [23,24]. The seedling stage is particularly vulnerable to abiotic stress; thus, early phenotyping can improve selection efficiency in breeding programs [25].

Despite extensive research on wheat responses to individual stresses, studies that simultaneously assess growth, photosynthetic performance, pigment stability, osmolyte accumulation, and antioxidant activity across multiple genotypes under both drought and salinity are scarce. To fill this gap, we evaluated six wheat genotypes at the seedling stage under controlled drought and salinity conditions, integrating morphological, physiological, and biochemical traits with multivariate analyses. This approach aimed to (i) identify seedling-stage morpho-physiological and biochemical key traits associated with relative stress resilience under drought and salinity and (ii) prioritize candidate genotypes for their potential as donors in breeding programs targeting environments affected by water scarcity and soil salinization, thereby supporting the development of wheat cultivars with enhanced tolerance to multiple abiotic stresses.

## 2. Results

### 2.1. Effect of Drought and Salinity Stress on Growth Related Traits

Both drought and salinity significantly reduced growth and biomass in all wheat genotypes compared with controls (Table 1). Shoot length (SL), root length (RL), shoot fresh and dry weight (SFW, SDW), and root fresh and dry weight (RFW, RDW) declined under stress, with drought causing the most severe reductions.

**Figure 1:**
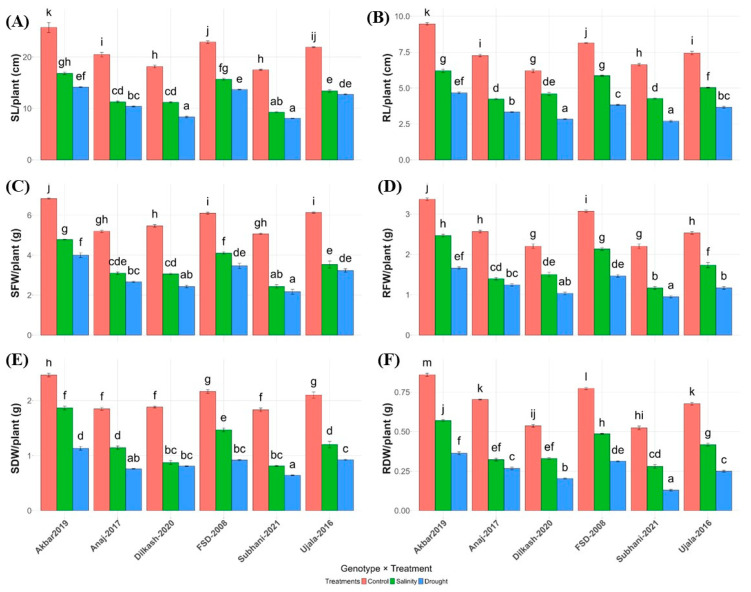
Influence of salinity and drought stress on various plant growth parameters in wheat varieties. (**A**) Shoot length (SL), (**B**) root length (RL), (**C**) shoot fresh weight (SFW), (**D**) root fresh weight (RFW), (**E**) shoot dry weight (SDW), and (**F**) root dry weight (RDW) per plant for different wheat varieties under control (orange), salinity (green), and drought (blue) conditions. Letters based on the least significant differences (LSD) test indicate significant differences among varieties within each treatment, with different letters representing significant variations (*p* < 0.05). Vertical lines at top of each bar illustrate standard error.

Among the genotypes, Akbar-2019 consistently maintained superior growth under both stresses, while Subhani-2021 was the most adversely affected (Figure 1). Under drought, SL and RL in Akbar-2019 decreased to 14.17 cm and 4.67 cm, respectively, compared with 8.07 cm and 2.70 cm in Subhani-2021. Under salinity, Akbar-2019 retained relatively higher SL (16.80 cm) and RL (6.20 cm), whereas Subhani-2021 remained the lowest (9.27 cm and 4.27 cm).

Biomass followed a similar pattern. Akbar-2019 had the highest SFW (6.83 g) and SDW (2.47 g) under control conditions, and maintained higher values under stress. Subhani-2021 recorded the lowest biomass, particularly under drought (SFW 2.17 g, SDW 0.64 g; RFW 0.95 g, RDW 0.13 g). Overall, Akbar-2019 demonstrated the greatest tolerance, whereas Subhani-2021 was the most sensitive across all growth traits and stress conditions.

### 2.2. Physiological Alterations Under Salinity and Drought Stress

**Figure 2:**
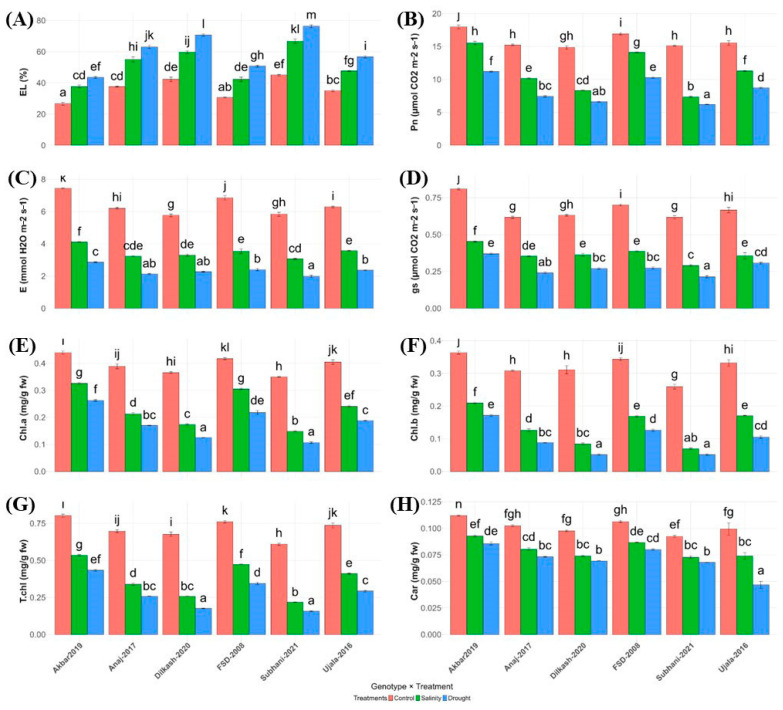
Influence of salinity and drought stress on physiological and biochemical parameters in wheat varieties. (**A**) Electrolyte leakage (EL), (**B**) net photosynthetic rate (Pn), (**C**) transpiration rate (E), (**D**) stomatal conductance (gs), (**E**) chlorophyll a (Chl.a), (**F**) chlorophyll b (Chl.b), (**G**) total chlorophyll (T.chl), and (**H**) carotenoid (Car) content of different wheat varieties under control (orange), salinity (green), and drought (blue) conditions. Letters based on the least significant differences (LSD) test indicate significant differences among varieties within each treatment, with different letters representing significant variations. (*p* < 0.05). Vertical lines at top of each bar illustrate standard error.

Both drought and salinity significantly affected physiological and biochemical traits in all wheat genotypes (Figure 2; Table 1). Electrolyte leakage (EL%), an indicator of membrane damage, increased under stress, with Subhani-2021 showing the highest values (66.67% under salinity, 76.33% under drought) and Akbar-2019 the lowest (37.67% and 43.50%), reflecting superior membrane stability (Figure 2A).

Photosynthetic performance declined under stress. Akbar-2019 maintained higher net photosynthetic rates (Pn: 17.97 µmol CO_2_ m^−2^ s^−1^ control; 15.53 salinity; 11.20 drought), whereas Subhani-2021 exhibited the strongest reduction (7.33 salinity; 6.20 drought) (Figure 2B). Similar patterns were observed for transpiration (E) and gs: Akbar-2019 retained higher values under both stresses, while Subhani-2021 showed the lowest, particularly under drought (E: 1.98 mmol H_2_O m^−2^ s^−1^; gs: 0.21 mol H_2_O m^−2^ s^−1^) (Figure 2C,D) respectively.

Chlorophyll and carotenoid contents were also reduced under stress. Akbar-2019 maintained higher total chlorophyll (T.chl mg g^−1^ FW: 0.803 control; 0.434 drought) and carotenoids (Car: 0.112 control), whereas Subhani-2021 showed the lowest pigment levels, with T.chl decreasing to 0.158 mg g^−1^ FW and Car to 0.068 mg g^−1^ FW under drought (Figure 2E–H). Overall, Akbar-2019 exhibited better maintenance of pigments and gas exchange under stress, while Subhani-2021 experienced pronounced pigment loss and impaired stomatal function.

### 2.3. Biochemical Changes Under Salinity and Drought Stress

**Figure 3:**
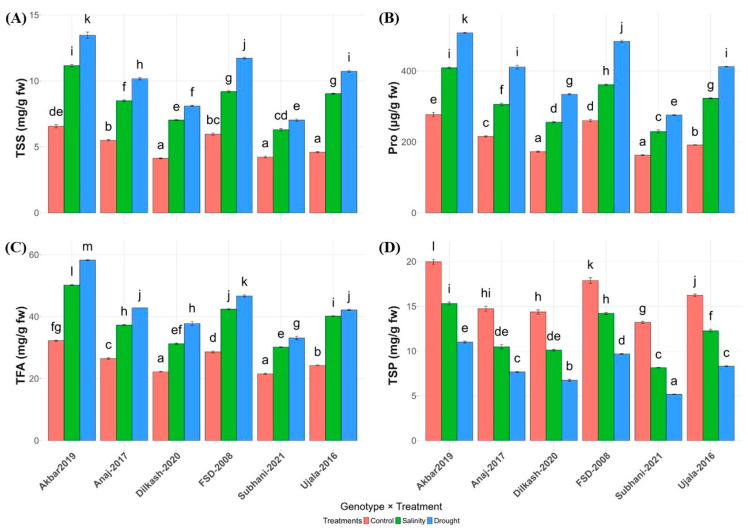
Influence of salinity and drought stress on biochemical markers in wheat varieties. (**A**) Total soluble sugars (TSSs), (**B**) proline (Pro), (**C**) total free amino acids (TFAs), and (**D**) total soluble protein (TSP) content of different wheat varieties under control (orange), salinity (green), and drought (blue) conditions. Letters based on the least significant differences (LSD) test indicate significant differences among varieties within each treatment, with different letters representing significant variations. (*p* < 0.05). Vertical lines at top of each bar illustrate standard error.

Salinity and drought significantly increased osmolytes, including total soluble sugars (TSSs), proline, and total free amino acids (TFAs), indicating osmotic adjustment under stress. Akbar-2019 showed the strongest response, recording the highest TSSs (11.17 mg g^−1^ FW under salinity; 13.47 mg g^−1^ FW under drought), proline (up to 507.63 µmol g^−1^ FW under drought), and TFAs (58.27 µmol g^−1^ FW under drought). In contrast, Subhani-2021 exhibited comparatively lower accumulation of TSSs, proline, and TFAs across treatments (Figure 3; Table 1).

Total soluble protein (TSP) declined under both stresses in all genotypes. However, Akbar-2019 maintained relatively higher protein content with moderate reductions, whereas Subhani-2021 showed the lowest values and the greatest decline under drought. Overall, Akbar-2019 demonstrated stronger biochemical adjustment under stress, while Subhani-2021 exhibited a comparatively weaker osmotic response (Figure 3).

### 2.4. Antioxidant Enzymes and Reactive Oxygen Species Production

**Figure 4:**
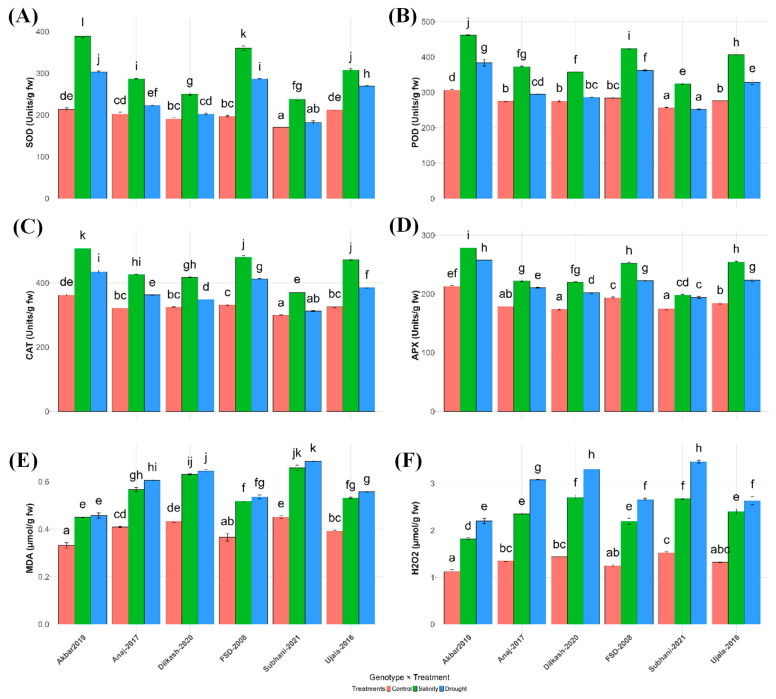
Influence of salinity and drought stress on antioxidant enzyme activities and oxidative stress markers in wheat varieties. (**A**) Superoxide dismutase (SOD), (**B**) peroxidase (POD), (**C**) catalase (CAT), (**D**) ascorbate peroxidase (APX), (**E**) malondialdehyde (MDA), and (**F**) hydrogen peroxide (H_2_O_2_) content of different wheat varieties under control (orange), salinity (green), and drought (blue) conditions. Letters based on the least significant differences (LSD) test indicate significant differences among varieties within each treatment, with different letters representing significant variations. (*p* < 0.05). Vertical lines at top of each bar illustrate standard error.

Salinity and drought substantially altered antioxidant defenses and oxidative stress markers (Figure 4; Table 2). Activities of SOD, POD, CAT, and APX increased under both stresses, although responses varied among genotypes.

Under control conditions, Akbar-2019 had the highest SOD activity (213.73 U mg^−1^), whereas Subhani-2021 had the lowest (170.57 U mg^−1^). Both stresses increased SOD, with Akbar-2019 reaching 389.17 U mg^−1^ under salinity and 303.50 U mg^−1^ under drought, indicating a stronger antioxidative response (Figure 4A). POD showed a similar trend: Akbar-2019 increased from 306.07 U mg^−1^ (control) to 462.27 U mg^−1^ (salinity) and 384.07 U mg^−1^ (drought), while Subhani-2021 remained lowest under stress (324.40 U mg^−1^ in salinity; 252.27 U mg^−1^ in drought) (Figure 4B).

CAT activity increased under both stresses, peaking in Akbar-2019 (362.30 U mg^−1^ in control; 507.63 U mg^−1^ under salinity; 434.80 U mg^−1^ under drought), whereas Subhani-2021 remained lowest, particularly under drought (312.60 U mg^−1^) (Figure 4C). APX showed a similar pattern: Akbar-2019 increased from 212.73 U mg^−1^ (control) to 278.43 U mg^−1^ (salinity) and 257.53 U mg^−1^ (drought), while Subhani-2021 exhibited the weakest response (193.97 U mg^−1^ under drought) (Figure 4D).

Malondialdehyde (MDA) and hydrogen peroxide (H_2_O_2_) significantly increased under both salinity and drought (Table 2). Akbar-2019 had the lowest baseline levels (MDA 0.332 µmol g^−1^; H_2_O_2_ 1.123 µmol g^−1^), whereas Subhani-2021 had the highest (0.452 and 1.527 µmol g^−1^, respectively) and showed the largest stress-induced increases, peaking under drought (MDA 0.686 µmol g^−1^; H_2_O_2_ 3.463 µmol g^−1^) (Figure 4E,F). Across treatments, Akbar-2019 maintained comparatively lower MDA and H_2_O_2_, whereas Subhani-2021 maintained higher values.

### 2.5. Principal Component Analysis

**Figure 5:**
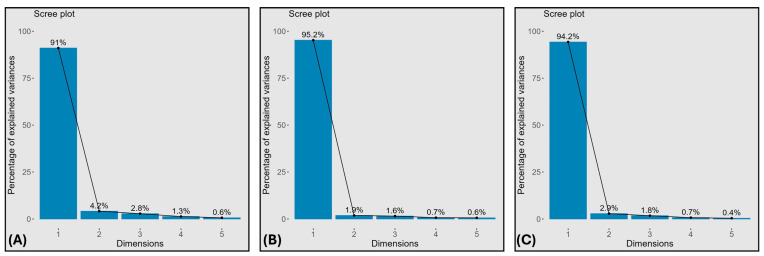
Scree plot analysis showcases the individual principal components scores (PCs) under (**A**) control, (**B**) salinity, and (**C**) drought stress conditions.

**Figure 6:**
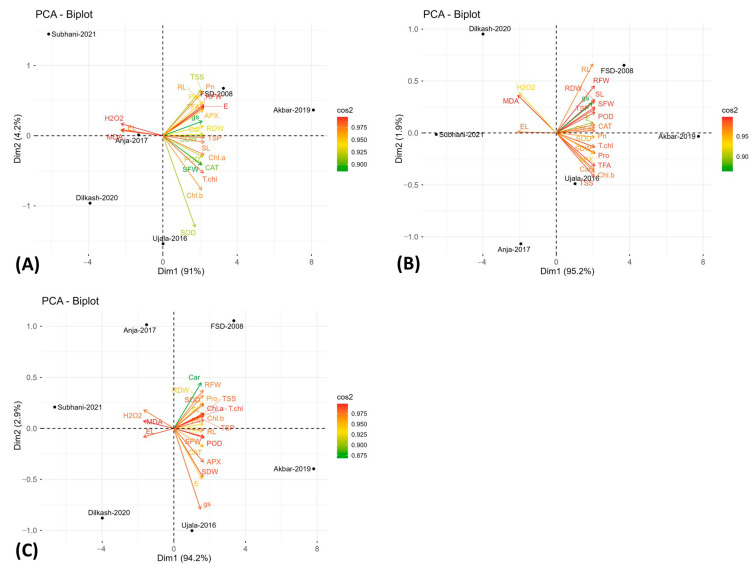
Principal component analysis for all the studied wheat genotypes under (**A**) control condition, (**B**) salinity stress, and (**C**) drought stress conditions to elucidate the variability patterns among all the analyzed traits and studied wheat accessions.

Principal component analysis (PCA) was used to summarize multivariate variation in morpho-physiological and biochemical traits under control (T_1_), salinity (T_2_), and drought (T_3_). Scree plots (Figure 5) showed that the first two components explained the largest share of variance across treatments and were therefore used for biplot interpretation (Figure 6, Table S2).

Across treatments, the first two principal components explained most of the variance (T_1_: PC1 91%, PC2 4.2%; T_2_: PC1 95.2%, PC2 1.9%; T_3_: PC1 94.2%, PC2 2.9%) (Figure 5A–C). In the biplots (Figure 6A–C), Akbar-2019 and FSD-2008 consistently clustered with growth- and performance-related traits, while damage-related variables loaded in the opposite direction. Under T_1_, Akbar-2019 and FSD-2008 aligned with TSSs, Pn, APX, RDW, Car, and E, whereas Anaj-2017 aligned with EL, H_2_O_2_, and MDA. Under salinity (T_2_), Akbar-2019 aligned with SDW, POD, CAT, SFW, Pn, Pro, and TSSs, and FSD-2008 aligned with RL, RFW, SL, g_s_, and TSP, while Subhani-2021 aligned with EL and Dilkash-2020 with H_2_O_2_ and MDA. Under drought (T_3_), Akbar-2019 aligned with RL, TSP, SFW, POD, CAT, and APX, and FSD-2008 aligned with RFW, RDW, SOD, Pro, and Car, whereas Dilkash-2020 and Subhani-2021 clustered with higher EL, H_2_O_2_, and MDA.

### 2.6. Heatmap Analysis

**Figure 7:**
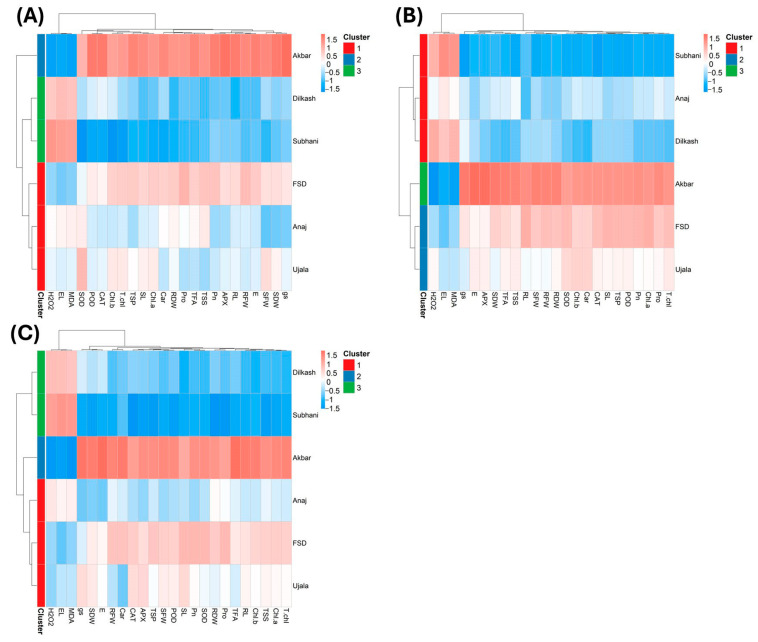
Heatmap analysis revealing positive and negative interactions among all the analyzed traits and clustering patterns of studied wheat genotypes under (**A**) control, (**B**) salinity, and (**C**) drought stress conditions.

Heatmap analysis was used to visualize genotype clustering and trait associations under control, salinity, and drought (Figure 7). Under control conditions (T_1_), three distinct clusters were identified (Figure 7A). Akbar-2019, within the second cluster (blue), clustered with higher Pn, TSSs, and RDW and showed negative associations with EL, H_2_O_2_, and MDA. Genotypes Dilkash-2020, Subhani-2021, and Anaj-2017 grouped in the third cluster (green), characterized by weaker associations with growth and antioxidant traits. The first cluster (red), comprising FSD-2008, Anaj-2017, and Ujala-2016, showed comparatively weaker associations with traits such as Car, T.chl, SFW, SOD, and MDA. Among all genotypes, Akbar-2019 performed best under optimal conditions.

Under salinity stress (T_2_), the heatmap again divided genotypes into three clusters (Figure 7B). The first cluster (red; Subhani-2021, Anaj-2017, and Dilkash-2020) aligned positively with EL, MDA, and H_2_O_2_ and negatively with growth and antioxidant traits. The second cluster (blue; Akbar-2019) aligned positively with Pn, TSSs, SDW, and RDW and negatively with oxidative stress markers. The third cluster (green; Ujala-2016 and FSD-2008) showed intermediate associations across traits.

Under drought (T_3_), genotypes again separated into three main clusters (Figure 7C). The first cluster (red; Anaj-2017, FSD-2008, and Ujala-2016) showed intermediate associations across growth, physiological, and biochemical traits. The second cluster (blue; Akbar-2019) aligned positively with Pn, TSSs, SDW, and RDW and negatively with EL, H_2_O_2_, and MDA. The third cluster (green; Dilkash-2020 and Subhani-2021) aligned negatively with growth and antioxidant traits and positively with EL, H_2_O_2_, and MDA.

### 2.7. Pearson’s Correlation Analysis

**Figure 8:**
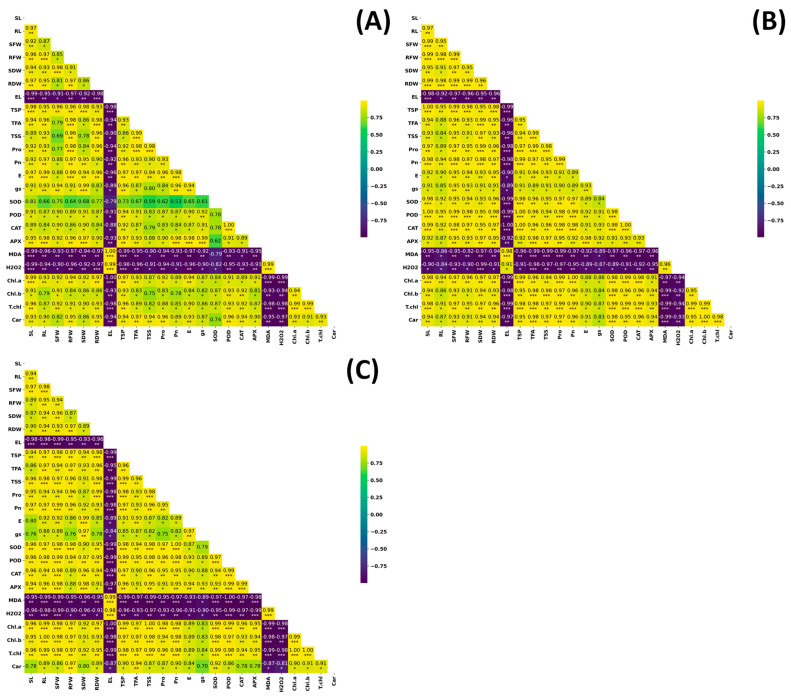
Pearson’s correlation analysis illustrates the significant positive and negative correlation among all the analyzed attributes under (**A**) control, (**B**) salinity, and (**C**) drought stress conditions. Asterisks divulge statistical significance levels (* *p* < 0.05, ** *p* < 0.01, *** *p* < 0.001).

Under control conditions (T_1_), Pearson’s correlation analysis showed tight coordination among growth traits (Figure 8A). SL and RL were strongly correlated with RDW (r = 0.98 ***), SFW (r = 0.94 ***), and SDW (r = 0.93 ***), indicating integrated biomass accumulation under optimal conditions. Antioxidant enzymes also covaried, with a strong association between SOD and POD (r = 0.91 ***), consistent with coordinated basal antioxidant activity. EL was strongly negatively correlated with TSP (r = −0.94 ***), linking membrane integrity with protein retention. Oxidative damage markers were inversely related to antioxidant capacity: MDA correlated negatively with SOD (r = −0.93 ***) and APX (r = −0.90 ***). Pigment variables were highly interrelated (e.g., Chl.a vs. T.chl, r = 0.96 ***), indicating coordinated regulation of chlorophyll composition.

Under salinity (T_2_), correlations among growth traits remained highly significant and positive (Figure 8B). SL and RL were highly correlated with RDW (r = 0.98 ***), SFW (r = 0.99 ***), and SDW (r = 0.97 ***), indicating coordinated growth responses under salinity. EL remained strongly negatively associated with TSP (r = −0.94 ***), linking membrane injury with reduced protein retention. Antioxidant enzymes covaried tightly, with SOD strongly correlated with POD (r = 0.99 ***), and MDA negatively correlated with SOD (r = −0.90 ***), consistent with an inverse relationship between lipid peroxidation and antioxidant capacity. Chlorophyll traits remained highly interrelated (Chl.a vs. T.chl, r = 0.96 ***).

Under drought (T_3_), the overall correlation structure was maintained but strengthened (Figure 8C). Growth traits remained tightly linked: SL and RL correlated strongly with RDW (r = 0.94 ***), SFW (r = 0.97 ***), and SDW (r = 0.94 ***). EL showed an even stronger negative association with TSP (r = −0.99 ***), indicating close coupling between membrane integrity and protein retention under drought. Antioxidant enzymes covaried strongly, with SOD and POD highly correlated (r = 0.99 ***). Oxidative stress markers were inversely related to antioxidant activity: MDA and H_2_O_2_ correlated negatively with SOD (r = −0.96 *** and r = −0.99 ***, respectively). Chlorophyll traits were also highly interrelated (Chl.a vs. T.chl, r = 0.98 ***), consistent with coordinated pigment responses. Collectively, Figure 8 indicates that growth, membrane stability, oxidative markers, antioxidant enzymes, and pigment traits are tightly interconnected across treatments.

### 2.8. Multi-Trait Genotype–Ideotype Distance Index

**Figure 9:**
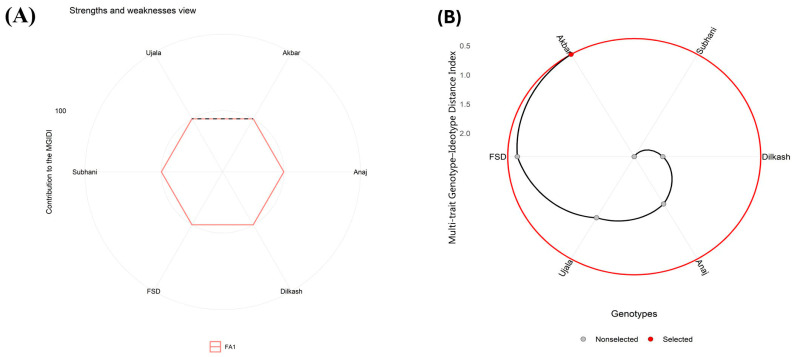
(**A**) Under the control condition, the Multi-trait Genotype–Ideotype Distance Index (MGIDI) analysis illustrates the relative contribution of each factor by highlighting genotype strengths and limitations. Factors positioned closer to the central region indicate that the associated traits are more consistent with the ideotype, whereas factors extending farther from the center reflect weaker performance for those trait groups in a given genotype. The dashed reference circle denotes the expected contribution, assuming all factors have equal weight. (**B**) Genotypes are ordered according to their overall performance across the evaluated traits, with the chosen genotype emphasized by a black trajectory and a red marker.

Under control conditions, the Multi-trait Genotype–Ideotype Distance Index (MGIDI) ranked Akbar-2019 closest to the ideotype, followed by FSD-2008 (Figure 9A,B). Ujala-2016 and Subhani-2021 showed intermediate performance, whereas Anaj-2017 and Dilkash-2020 ranked lowest. In contrast, Anaj-2017 and Dilkash-2020 exhibited factor contributions extending farther from the center, indicating weaker performance for the corresponding trait groups. Overall, MGIDI ranked Akbar-2019 closest to the ideotype, followed by FSD-2008, while Ujala-2016 and Subhani-2021 occupied intermediate positions that may merit further evaluation for trait improvement.

**Figure 10:**
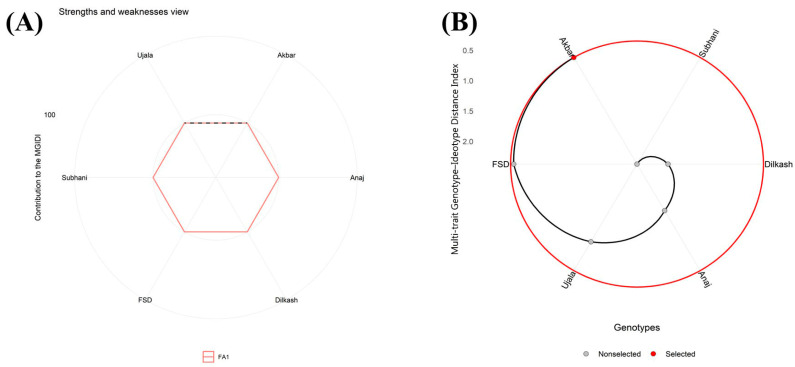
(**A**) Under salinity stress (100 mM NaCl), the Multi-trait Genotype–Ideotype Distance Index (MGIDI) analysis illustrates the relative contribution of each factor by highlighting genotype strengths and limitations. Factors positioned closer to the central region indicate that the associated traits are more consistent with the ideotype, whereas factors extending farther from the center reflect weaker performance for those trait groups in a given genotype. The dashed reference circle denotes the expected contribution, assuming all factors have equal weight. (**B**) Genotypes are ordered according to their overall performance across the evaluated traits, with the chosen genotype emphasized by a black trajectory and a red marker.

Under salinity stress, MGIDI ranked Akbar-2019 closest to the ideotype, followed by FSD-2008 (Figure 10A,B). Ujala-2016 showed intermediate performance, whereas Subhani-2021, Dilkash-2020, and Anaj-2017 ranked lower (Figure 10). The lower-ranked genotypes showed factor contributions extending farther from the center, indicating weaker performance within the corresponding trait groups under salinity (Figure 10). Overall, Akbar-2019 exhibited the strongest multi-trait profile under salinity, with FSD-2008 and Ujala-2016 occupying intermediate positions and Dilkash-2020 and Anaj-2017 showing the greatest divergence from the ideotype.

**Figure 11:**
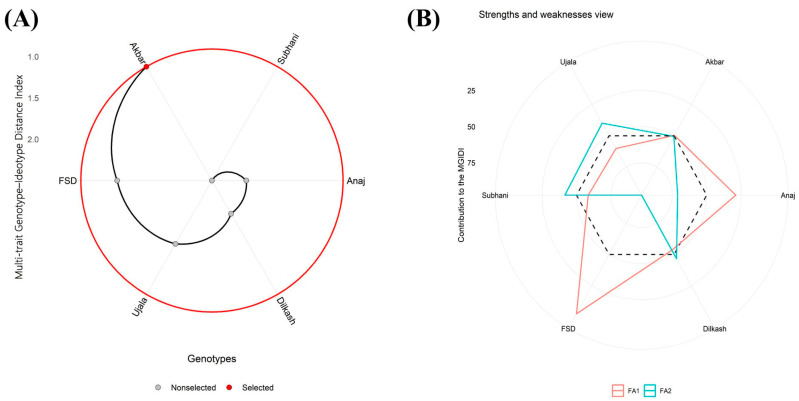
(**A**) Under drought stress (FC 50%), the Multi-trait Genotype–Ideotype Distance Index (MGIDI) analysis illustrates the relative contribution of each factor by highlighting genotype strengths and limitations. Factors positioned closer to the central region indicate that the associated traits are more consistent with the ideotype, whereas factors extending farther from the center reflect weaker performance for those trait groups in a given genotype. The dashed reference circle denotes the expected contribution assuming all factors have equal weight. (**B**) Genotypes are ordered according to their overall performance across the evaluated traits, with the chosen genotype emphasized by a black trajectory and a red marker.

Under drought stress, MGIDI ranked Akbar-2019 and FSD-2008 closest to the ideotype, with their profiles driven largely by FA1 (growth-related traits) (Figure 11A,B). FA2 (physiological traits) contributed more strongly to Ujala-2016, Dilkash-2020, and Subhani-2021, placing these genotypes closer to the ideotype for that trait group. In contrast, Dilkash-2020 and Anaj-2017 showed weaker overall profiles, with factor contributions extending farther from the center across growth and physiological trait groups (Figure 11A,B). Overall, Akbar-2019 emerges as the most resilient genotype under drought stress and is considered a promising candidate for breeding programs focused on drought tolerance. Conversely, Anaj-2017 shows the lowest performance, indicating the greatest divergence from the ideotype.

## 3. Discussion

The present study integrates morphological, physiological, biochemical, and antioxidant responses of six wheat genotypes at the seedling stage under salinity and drought stress to identify traits associated with stress resilience. Both stress factors significantly suppressed shoot and root growth as well as biomass accumulation; however, the extent of reduction varied considerably among genotypes. Akbar-2019, followed by FSD-2008, maintained comparatively greater shoot and RL along with higher fresh and dry biomass (Figure 1), whereas Subhani-2021 and Dilkash-2020 exhibited pronounced growth inhibition. Drought stress imposed stronger growth reductions than salinity, which is consistent with previous reports indicating that osmotic imbalance and ionic disturbances restrict water uptake, cell expansion, and overall plant growth under abiotic stress conditions [26,27]. Similar reductions in growth have been widely reported in plants exposed to salinity and drought due to limitations in water availability, metabolic disruption, and energy diversion toward stress defense mechanisms [10,28].

Multi-trait assessment using the MGIDI further supported these genotype-dependent responses, where Akbar-2019 consistently ranked closest to the ideotype under all treatments (Figure 9, Figure 10 and Figure 11). The intermediate positioning of other genotypes suggests that tolerance was governed by a combination of multiple physiological and biochemical traits rather than a single determinant factor. Such variability among genotypes is expected because plants differ in their capacity for osmotic adjustment, metabolic regulation, and ion homeostasis under stress conditions [7,8]. Effective regulation of Na^+^ and K^+^ balance and cellular ion compartmentalization are key determinants of salinity tolerance, allowing plants to maintain enzymatic activity and metabolic stability even under elevated salt concentrations [11]. In contrast, the relatively poor ranking of Subhani-2021 and Dilkash-2020 reflects their limited capacity to sustain growth and protective responses, which is commonly associated with increased susceptibility to abiotic stresses [29].

Salinity and drought also markedly impaired physiological processes related to gas exchange and membrane stability (Figure 2). Electrolyte leakage increased substantially in Subhani-2021, indicating severe membrane damage, whereas Akbar-2019 maintained comparatively lower leakage, suggesting improved membrane protection and structural stability. Reductions in photosynthetic rate, gs, and E were observed in all genotypes, but the decline was less pronounced in Akbar-2019, which retained higher CO_2_ assimilation capacity and overall physiological activity under stress [30,31]. These responses are consistent with previous findings showing that both salinity and drought limit photosynthesis primarily through stomatal closure, reduced mesophyll conductance, and metabolic constraints in the photosynthetic apparatus [7,10].

Photosynthetic pigments including chlorophyll a, chlorophyll b, total chlorophyll, and carotenoids also declined significantly under both stress treatments, with drought causing the greatest reductions (Figure 2E–H). However, Akbar-2019 maintained relatively higher pigment concentrations compared with the sensitive genotypes, indicating better preservation of the photosynthetic machinery. Stress-induced pigment loss is frequently associated with oxidative damage and disruption of chloroplast structure caused by reactive oxygen species (ROS) accumulation [32]. ROS overproduction under abiotic stress conditions can damage lipids, proteins, and nucleic acids, thereby impairing photosynthetic efficiency and cellular metabolism [17,18].

Accumulation of compatible solutes such as TSSs (Figure 3A), proline (Figure 3B), and total free amino acids (Figure 3C) increased markedly in response to both stresses, reflecting the activation of osmotic adjustment mechanisms (Figure 3). Akbar-2019 exhibited the greatest accumulation of these osmolytes, which likely contributed to improved cellular water retention, stabilization of proteins and membranes, and protection of metabolic processes under stress. Conversely, Subhani-2021 displayed comparatively lower osmolyte accumulation, indicating weaker osmotic regulation capacity. TSP levels (Figure 3D) declined under stress, but the reduction was less severe in Akbar-2019, suggesting improved protection of metabolic enzymes and cellular structures [33]. The accumulation of osmoprotectants is widely recognized as a critical adaptive strategy enabling plants to maintain turgor pressure and metabolic activity during periods of osmotic stress (Figure 3) [12,28].

Antioxidant defense mechanisms also played a crucial role in differentiating tolerant and sensitive genotypes. Akbar-2019 demonstrated significantly higher activities of key antioxidant enzymes including SOD, POD, CAT, and APX (Figure 4), accompanied by lower levels of oxidative stress indicators such as MDA and H_2_O_2_. These responses indicate a more efficient detoxification system capable of controlling ROS accumulation and preventing oxidative damage. In contrast, Subhani-2021 exhibited relatively lower antioxidant enzyme activity and greater oxidative damage, suggesting a weaker redox defense system. The coordinated functioning of antioxidant enzymes is essential for maintaining cellular redox balance, as SOD converts superoxide radicals to H_2_O_2_, which is subsequently detoxified by CAT and APX [17,18]. Effective ROS scavenging is therefore critical for protecting cellular structures and sustaining metabolic processes under stress conditions [34].

Although both salinity and drought impose osmotic stress that restricts water uptake, the severity of their physiological effects may differ. In the present study, drought stress at 50% field capacity likely resulted in a more direct and sustained reduction in soil water availability, leading to stronger limitations on cell expansion, turgor maintenance, and carbon assimilation [35]. Under salinity conditions, plants initially experience osmotic stress, but may partially adapt through osmolyte accumulation and ion compartmentalization, which can help maintain cellular water balance and metabolic activity [8,11]. In contrast, progressive soil moisture depletion under drought can intensify stomatal closure, reduce CO_2_ diffusion into leaves, and consequently decrease photosynthetic carbon fixation, ultimately resulting in greater biomass reduction [36]. These physiological responses align with broader syntheses of plant adaptation to water deficit and osmotic stress [28,37].

Multivariate analyses provided further support for these interpretations. PCA clearly separated tolerant genotypes (Akbar-2019 and FSD-2008) from sensitive genotypes (Subhani-2021 and Dilkash-2020), with tolerance associated with growth traits, photosynthetic performance, osmolyte accumulation, and antioxidant activity, while susceptibility corresponded with oxidative damage indicators and membrane instability (Figure 6). Heatmap clustering produced a similar grouping pattern, and correlation analysis revealed strong positive relationships among growth attributes and antioxidant enzyme activities, while oxidative stress markers (MDA and H_2_O_2_) were negatively correlated with antioxidant responses. These results highlight the integrative nature of plant stress tolerance, where multiple physiological and biochemical processes operate together to maintain cellular homeostasis [38]. Previous studies have also emphasized the value of heatmap clustering in identifying genotype groupings and understanding trait interactions in stress tolerance studies [39,40]. Moreover, MGIDI rankings under control (Figure 9), salinity (Figure 10), and drought stress (Figure 11) closely aligned with PCA (Figure 6) and heatmap (Figure 7) outputs, confirming Akbar-2019, followed by FSD-2008, as the genotypes most closely matching the ideotype across treatments, consistent with earlier demonstrations of MGIDI’s effectiveness for multi-trait genotype evaluation [41].

Overall, the combined physiological, biochemical, and statistical evidence indicates that the superior stress tolerance of Akbar-2019 results from the integration of several adaptive mechanisms, including efficient osmotic adjustment, maintenance of photosynthetic pigments and gas exchange, effective ion regulation, and strong antioxidant defense systems that limit ROS-mediated cellular damage (Figure 2, Figure 3 and Figure 4). In contrast, the susceptibility of Subhani-2021 was associated with weaker osmolyte accumulation, greater pigment degradation, increased membrane injury, and insufficient antioxidant activation. These contrasting physiological profiles illustrate the complex interplay of metabolic, biochemical, and structural factors that collectively determine stress tolerance in wheat [42].

The results show the convergence of growth measurements, biochemical profiling, and multivariate analyses supports the prioritization of Akbar-2019, followed by FSD-2008, as promising germplasm for breeding programs aimed at improving wheat tolerance to salinity and drought. Nevertheless, genotype performance can vary across developmental stages, particularly during reproductive phases such as flowering, anthesis, and grain filling, which are highly sensitive to environmental stress. Because the present evaluation was conducted at the seedling stage under controlled conditions, further validation across growth stages and field environments is required. Despite this limitation, previous studies suggest that wheat genotypes exhibiting strong vigor during early growth stages often display improved tolerance at later developmental stages, indicating that seedling-stage screening can serve as a useful preliminary tool for identifying stress-resilient genotypes.

## 4. Materials and Methods

The present study was carried out in the greenhouse of the Plant Stress Physiology Lab., NIAB, Faisalabad, Pakistan, in collaboration with the Department of Botany, University of Lahore, Pakistan, to assess the physio-biochemical basis of salinity and drought tolerance in wheat (*Triticum aestivum* L.) at the seedling stage. Certified seeds of six commercially important wheat cultivars, including Anaj-2017, FSD-2008, Akbar-2019, Ujala-2016, Dilkash-2020, and Subhani-2021, were obtained from the Ayub Agriculture Research Institute (AARI), Pakistan Agriculture Research Council (PARC), Faisalabad, Pakistan. These genotypes were chosen based on their contrasting performance reported in previous studies and local cultivation history. Such as Akbar-2019 and Subhani-2021, have been identified as relatively tolerant and sensitive to abiotic stresses, respectively [43], while others represent widely cultivated and agronomically important varieties. The selection also aimed to capture diversity in physiological and morphological traits relevant to stress response, allowing a comprehensive comparative evaluation under both drought and salinity conditions.

A container-based study was conducted in a glasshouse of NIAB, Faisalabad, under controlled conditions to evaluate the physio-morphic and biochemical markers of stress tolerance under semi-natural soil conditions. The experiment followed a completely randomized design (CRD) with 3 biological replicates per wheat genotype in each treatment. Plastic pots (20 cm diameter and 15 cm length) were filled with 3 kg sterilized mixture of alluvial soil and washed river sand (3:1, *v*/*v*) to ensure adequate aeration and drainage. Soil properties were pH 7.4, EC 1.2 dS m^−1^, and organic matter 0.9%. Ten seeds per pot were sown and thinned to five uniform seedlings after emergence. After one week, drought stress was imposed by maintaining pots at 50% field capacity, as described by Barrs and Weatherley [44]. For the salinity treatment, plants were irrigated with 100 mM NaCl after two weeks (14 days) of establishment. Control plants received distilled water. Both experiments were maintained at a temperature of 28 ± 2 °C with a 12 h photo period.

### 4.1. Maintenance of Field Capacity (FC)

For the maintenance of 100% field capacity (100% FC) standard gravimetric method as described by Weatherley [45] was used. First, the soil was air-dried and passed through a 2 mm sieve to ensure uniform texture. The dry weight of each empty pot (W_1_) was recorded. Three kilograms (kg) of dry soil was then added to each pot, and the combined weight of the pot plus the dry soil was recorded (W_2_). To determine field capacity, distilled water was slowly added to each pot until the soil became fully saturated and water just began to drain from the bottom. The pots were then covered to minimize evaporation and allowed to drain freely for 24–48 h at room temperature. After drainage had ceased, each pot was weighed again; this weight was considered the weight at 100% field capacity (W_FC_). The amount of water retained at field capacity was calculated as W_FC_−W_2_.

To establish 50% field capacity, the target pot weight was calculated as:

Target weight at 50% FC = W_2_ + 0.5 × (W_FC_ − W_2_).

This calculated value served as the reference weight for maintaining the drought treatment. During the experimental period, pots were weighed daily (after 24 h intervals), and water loss due to evapotranspiration was determined gravimetrically. The required amount of distilled water was added to each pot to restore its weight to the predetermined 50% FC level. This approach ensured that soil moisture was consistently maintained at 50% of field capacity throughout the stress period. Throughout the experiment, the pot, not the individual plant, was considered as the experimental unit. All pots were filled with equal amounts of soil to maintain uniform water-holding capacity and drainage characteristics. Soil moisture levels were monitored regularly to avoid deviations from the targeted 50% field capacity.

### 4.2. Salinity Treatment

Salinity stress was imposed using a 100 mM NaCl solution. To avoid sudden osmotic shock to the seedlings, the final concentration was achieved in two equal steps, with 50 mM NaCl applied per dose at consecutive irrigations. The saline solution was applied at a fixed irrigation interval, and a measured volume was added to each pot to ensure uniform distribution of salts in the soil. Throughout the treatment period, the electrical conductivity (EC) of the growth substrate was monitored periodically to confirm that the intended salinity level was established and maintained consistently.

### 4.3. Morphological Attributes

Two weeks after stress imposition, SL, RL, shoot fresh weight (SFW), root fresh weight (RFW), shoot dry weight (SDW), and root dry weight (RDW) were recorded. Shoot and root length were measured (cm) using a meter scale. Fresh mass of shoot and root tissues was measured using an electronic balance (model FA2104B). Tissues were then oven-dried at 70 °C for four days (YPO-072) to constant mass and re-weighed.

### 4.4. Physiological Analysis

Net photosynthetic rate (Pn), E, and gs were measured using a leaf-level infrared gas analyzer (CI-340, CID Inc., Camas, WA, USA) under control, salinity (100 mM NaCl), and drought (50% field capacity) conditions. Measurements were collected on sunny days between 09:30 and 12:00 h on the youngest fully expanded leaf of each replicate after readings stabilized. During measurements, ambient CO_2_ concentration, molar air flow per unit leaf area (403.3 mmol m^−2^ s^−1^), atmospheric pressure (99.9 kPa), and water vapor pressure (6.0–8.9 mbar) were maintained in the chamber. Photosynthetically active radiation at the leaf surface ranged from 1150 to 1300 μmol m^−2^ s^−1^, leaf temperature from 28 to 30 °C, and ambient 32 °C.

### 4.5. Electrolyte Leakage

Electrolyte leakage was quantified from fully expanded flag leaves using the method described by Lutts [12] and frameworks established for oxidative stress studies established by Velikova [46]. Briefly, chopped leaf tissue (1 g) from each genotype was rinsed three times with deionized water and incubated in 10 mL deionized water for 24 h at 5–15 °C. Initial electrical conductivity (EC_1_) was recorded using a calibrated conductivity meter (Mettler Toledo Seven Compact, Singapore). Samples were then autoclaved (DGL-50G) at 121 °C for 15 min, cooled to room temperature, and the final conductivity (EC_2_) was recorded. Electrolyte leakage (%) was calculated as:

*Electrolyte leakage* (%) = (*EC*_1_/*EC*_2_) × 100

### 4.6. Biochemical Analyses

Pro was determined using the ninhydrin assay described by Bates [47]. Fresh leaf tissue (0.5 g) was homogenized in 3% sulfosalicylic acid, and the extract was reacted with acid ninhydrin and glacial acetic acid. The resulting chromophore was extracted with toluene, and absorbance was measured at 520 nm using a UV–Vis spectrophotometer (U-2900, Hitachi High-Tech Corporation, Minato-ku, Tokyo, Japan). Pro content was calculated using a standard curve prepared with analytical-grade proline. TSSs were quantified using the anthrone reagent method established by Yemm and Willis [48] and modified by Raizi [49]. Dried leaf samples (0.1 g) were extracted in 80% ethanol, and 1 mL of extract was mixed with 5 mL of anthrone reagent. The mixture was heated in a boiling water bath for 10 min, cooled, and the absorbance was measured at 625 nm; glucose served as the standard. TFAs were estimated using the method described by Hamilton [50]. The reaction mixture containing 2% ninhydrin reagent and 10% pyridine was heated with the leaf extract at 95 °C for 30 min. After cooling, absorbance was recorded at 570 nm, and leucine served as the calibration standard. TSP was determined using the Lowry method [51] with normalization based on Bradford (1976) standards [52]. Fresh leaf tissue (0.2 g) was homogenized in 5 mL of 0.2 M phosphate buffer (pH 7.0). Absorbance was read at 620 nm, and protein content was estimated from a standard curve prepared with bovine serum albumin (BSA).

### 4.7. Antioxidant Enzyme Activity

Antioxidant enzymes were assayed using fully developed flag leaves to characterize stress-induced changes in enzymatic defense. SOD activity was measured based on inhibition of the photochemical reduction in nitroblue tetrazolium (NBT) in the presence of superoxide radicals. The reaction mixture consisted of plant extract, riboflavin, NBT, phosphate buffer, and methionine, and absorbance was recorded at 560 nm [53]. POD activity was determined using a guaiacol-based assay in which the enzyme catalyzes oxidation of guaiacol in the presence of H_2_O_2_; the change in absorbance was measured at 470 nm. CAT activity was determined by monitoring the rate of H_2_O_2_ breakdown; the reaction mixture included plant extract and H_2_O_2_ in phosphate buffer, and the reduction in absorbance was measured at 240 nm [54]. APX activity was evaluated by the decrease in absorbance at 290 nm as H_2_O_2_ oxidizes ascorbate in the presence of the plant extract [55]. MDA, a marker of lipid peroxidation, was measured by the thiobarbituric acid reactive substances (TBARS) assay; extracts were mixed with thiobarbituric acid reagent, heated, and the absorbance of the MDA–TBA complex was recorded at 532 nm [56]. H_2_O_2_ was determined by reacting plant extract with potassium iodide (KI) in an acidic medium, with color intensity measured at 390 nm [46].

### 4.8. Statistical Analyses

A two-factor factorial design analyzed using two-way ANOVA, including the main effects of genotype (G), treatment (T), and their interaction (G × T). Tukey’s HSD test was conducted in R (version 4.5.2) to evaluate differences among treatments and genotypes. PCA was used to summarize multivariate patterns across physio-morphic and biochemical traits and to identify trait associations among genotypes. PCA was conducted using FactoMineR and factoextra packages in R [53]. Heatmap analysis was performed in R using the *pheatmap* package to visualize trait relationships and to cluster genotypes based on their response patterns under control, salinity, and drought conditions [54]. Prior to clustering, data were standardized (mean-centered and scaled to unit variance) to ensure comparability among traits measured in different units. Hierarchical clustering was conducted using Euclidean distance and the complete linkage method.

The Multi-trait Genotype–Ideotype Distance Index (MGIDI) was computed separately for each treatment (control, salinity, and drought) using the *metan* package in R [55]. For each treatment, genotype means were used as input. Traits were first standardized, and desirability directions were defined according to biological relevance (e.g., higher values favored for growth, yield, and stress-tolerance traits; lower values favored for stress injury indicators). Traits requiring minimization were automatically rescaled within the MGIDI procedure; no additional manual inversion was applied. Equal weights were assigned to all traits. Factor analysis was conducted as implemented in the *metan* package, with factor retention based on eigenvalues > 1. The ideotype was defined separately for each treatment, reflecting the optimal trait profile under that specific condition. Selection intensity was set at the default level provided in the package, and the top-ranked genotypes were identified based on the lowest MGIDI values.

## 5. Conclusions

Under controlled glasshouse conditions, clear genotypic variation was observed in seedling-stage responses to salinity and drought stress. Among the tested genotypes, Akbar-2019 (followed by FSD-2008) demonstrated comparatively greater stability in growth attributes, pigment retention, osmotic adjustment, and antioxidant activity, together with relatively lower indicators of oxidative damage. In contrast, Subhani-2021 and Dilkash-2020 exhibited weaker physiological and biochemical adjustment, reflected in greater pigment degradation, reduced membrane stability, and elevated oxidative stress markers. These findings indicate that integrated trait-based screening, supported by multivariate analyses and MGIDI, can discriminate among genotypes at the early growth stage under controlled stress conditions. The putatively tolerant genotype(s) identified here are intended for early-stage screening and hypothesis generation and should not be construed as direct predictors of reproductive-stage yield performance without field validation. These candidates warrant follow-up in multi-location field trials and at later developmental stages. Collectively, our integrative physiological and biochemical analyses support a coordinated osmotic–photochemical–redox resilience signature at the seedling stage and motivate subsequent field-based and targeted mechanistic testing rather than direct cultivar recommendations.

## Figures and Tables

**Table 1:** Analysis of variance (ANOVA) for the effects of genotype (V) salinity and drought stress (T), along with their interactions, on morphological, physiological, and biochemical traits.

Source	DF	SL	RL	SFW	RFW	SDW	RDW	EL	TSP	TFAs	TSSs	Pro	Pn	E	gs	Phen
V	5	72.66 ***	6.97 ***	4.75 ***	1.46 ***	0.63 ***	0.1001 ***	908.25 ***	52.39 ***	397.25 ***	24.83 ***	39,351 ***	41.005 ***	1.63 ***	0.03 ***	16,497.6 ***
T	2	501.79 ***	73.99 ***	40.21 ***	9.14 ***	6.63 ***	0.836 ***	2644.49 ***	286.66 ***	1482.93 ***	118.62 ***	164,150 ***	261.51 ***	79.01 ***	0.77 ***	13,631.7 ***
V × T	10	1.251 ***	0.37 ***	0.08 ***	0.07 ***	0.05 ***	0.0046 ***	24.86 ***	0.58 ***	21.2 ***	1.86 ***	1732 ***	3.318 ***	0.14 ***	0.0015 ***	66,837.6 ***
Error	36	0.261	0.02	0.02	0.005	0.002	0.0002	2.67	0.09	0.24	0.024	25	0.089	0.0149	0.0002	21.9
Total	53															

Traits analyzed are shoot length (SL), root length (RL), shoot fresh weight (SFW), root fresh weight (RFW), shoot dry weight (SDW), root dry weight (RDW), electrolyte leakage (EL), total soluble proteins (TSP), total free amino acids (TFAs), total soluble sugars (TSSs), proline content (Pro), net photosynthesis (Pn), transpiration rate (E), stomatal conductance (gs), and phenolic contents (Phen). The levels of significance are depicted as *** *p* < 0.001.

**Table 2:** Analysis of variance (ANOVA) for the effects of genotype (V), salinity and drought (T), along with their interactions, on photosynthetic pigments and enzymatic activity.

Source	DF	Chl.a	Chl.b	T.chl	Car	SOD	POD	CAT	APX	MDA	H_2_O_2_
V	5	0.02637 ***	0.01734 ***	0.08486 ***	0.00077 ***	14,598.9 ***	12,934.4 ***	12,768.5 ***	4225.9 ***	0.04 ***	0.86 ***
T	2	0.22386 ***	0.24728 ***	0.94332 ***	0.00227 ***	52,208.4 ***	58,168.4 ***	12,030.1 ***	63,430.6 ***	0.18481 ***	11.21 ***
V × T	10	0.00121 ***	0.00067 ***	0.00295 ***	0.00023 ***	2081.7 ***	1277.1 ***	983.2 ***	243.7 ***	0.00135 ***	0.099 ***
Error	36	0.00006	0.00006	0.0002	0.00005	25.8	27.6	20.2	6.9	0.00015	0.0052
Total	53										

Traits analyzed include chlorophyll a (Chl.a), chlorophyll b (Chl.b), total chlorophyll (T.chl), carotenoid contents (Car), superoxide dismutase (SOD), peroxidase activity (POD), catalase activity (CAT), ascorbate peroxidase activity (APX), malondialdehyde (MDA), hydrogen peroxide (H_2_O_2_). The levels of significance are depicted as *** *p* < 0.001.

## Data Availability

The original contributions presented in this study are included in the article/Supplementary Material. Further inquiries can be directed to the corresponding authors.

## Supplementary Materials

The following supporting information can be downloaded at: https://www.mdpi.com/article/10.3390/plants15060930/s1, Table S1: Relationship of factors with traits, including original value (Xo), selected value (Xs), selection differential (SD), percentage of selection differential (SD%), heritability (h^2^), selection gains (SG), and percentage of selection gain (SG%) under conditions of control, moderate drought stress, and severe drought stress. Table S2: Factor loadings for each principal component under all applied treatments.
